# Efficacy of acupuncture in the management of ankylosing spondylitis: a systematic review and meta-analysis with insights

**DOI:** 10.3389/fneur.2025.1716550

**Published:** 2026-01-13

**Authors:** Junning Zhang, Shuchang Sun, Ruitong Bai, Hechun Yin, Jiawen Chen, Weiping Kong

**Affiliations:** 1 Graduate School, Beijing University of Chinese Medicine, Beijing, China; 2 Department of Traditional Chinese Medicine Rheumatology, China-Japan Friendship Hospital, Beijing, China; 3 School of Acupuncture-Moxibustion and Tuina, Beijing University of Chinese Medicine, Beijing, China; 4 Dongfang Hospital, Beijing University of Chinese Medicine, Beijing, China

**Keywords:** acupuncture, ankylosing spondylitis, complementary and alternative therapy, RCT, systematic review

## Abstract

**Background:**

Ankylosing spondylitis (AS) is a chronic, progressive autoimmune disease characterized by inflammation of the axial skeleton. Although acupuncture has been investigated as a potential therapeutic option for symptom relief, its efficacy in AS remains inconclusive. This systematic review and meta-analysis aimed to evaluate the efficacy and safety of acupuncture for patients with AS, aiming to generate evidence to inform clinical practice.

**Methods:**

This study was conducted in accordance with the Preferred Reporting Items for Systematic Reviews and Meta-Analyses (PRISMA) 2020 statement. A literature search was performed across 7 databases to identify articles published up to December 25, 2024. Only randomized controlled trials (RCTs) that met the eligibility criteria were included. The methodological quality of the included trials was assessed using the Cochrane Risk of Bias Tool (RoB 2.0), and meta-analyses were conducted with RevMan 5.4 software. If high heterogeneity was observed among the included studies, a random-effects model was used for data synthesis. Subgroup analyses and sensitivity analyses were performed when necessary.

**Results:**

A total of 35 randomized controlled trials (RCTs) met the inclusion criteria of this study. We used 6 indicators to estimate the results of the meta-analysis, all outcomes showed statistically significant improvements, including BASDAI score, BASFI score, ASDAS, VAS score, CRP level, and ESR level. Specifically, the BASDAI score (MD = −1.55, 95% CI: −1.97 to −1.13, *p* < 0.0001), BASFI score (MD = −1.47, 95% CI: −1.76 to −1.19, *p* < 0.0001), and ASDAS (MD = −0.57, 95% CI: −0.82 to −0.33, *p* < 0.0001). VAS score (MD = −1.30, 95% CI: −1.54 to −1.06, *p* < 0.0001), CRP level (MD = −7.12, 95% CI: −8.68 to −5.56, *p* < 0.0001), and ESR level (MD = −7.22, 95% CI: −9.13 to −5.32, *p* < 0.0001) all decreased significantly. Moreover, no serious acupuncture-related adverse events were reported across the 35 RCTs (only mild, transient reactions in a few cases).

**Conclusion:**

Acupuncture (including electroacupuncture, warm acupuncture, and fire acupuncture, etc.) is an effective and safe treatment for AS. However, the evidence base is limited by substantial heterogeneity and the lack of large-scale, high-quality clinical trials. Future research should focus on high-quality multicenter studies and comparative analyses of different acupuncture modalities to optimize the evidence for acupuncture in AS treatment.

**Systematic review registration:**

https://www.crd.york.ac.uk/PROSPERO/, identifier CRD42025633263.

## Introduction

Ankylosing spondylitis (AS) is an inflammatory rheumatic disease that affects the axial skeleton and is characterized by inflammatory back pain (1, 66). AS is one of the five spondyloarthritis subtypes (the others: psoriatic arthritis, reactive arthritis, spondyloarthritis with inflammatory bowel disease, undifferentiated spondyloarthritis) and is classified as a rheumatic immune disease (2). AS has an insidious onset and lacks specific symptoms in the early stage (3). With the progression of sacroiliac joint and axial skeleton inflammation, patients gradually develop chronic inflammatory low back pain, accompanied by morning stiffness and fatigue (4). Worsening spinal inflammation causes spinal stiffness and reduced mobility. Some patients may also have hip, knee, ankle, or shoulder swelling; the eyes are often involved, such as in acute anterior uveitis (5). AS affects approximately 0.1–0.5% of the global population, primarily young adults around 26 years old, with a higher prevalence in males (6).

The etiology of this disease remains unclear, but it exhibits obvious familial aggregation and a strong genetic association, with HLA-B27 serving as a key susceptibility gene (6, 7). At present, there is no clear cure for the disease, and clinical management focuses on controlling inflammation, relieving acute symptoms, and improving spinal function (8). The commonly used western medicines are non-steroidal anti-inflammatory drugs (NSAIDs) and disease-modifying antirheumatic drugs (DMARDs) (9, 10). However, current western therapies for AS only alleviate symptoms, requiring long-term use with high relapse risks, heavy economic burden, and notable side effects (11): long-term NSAIDs increase gastrointestinal, cardiovascular, and renal damage risks; conventional DMARDs act slowly with limited efficacy on axial symptoms (4, 65).

In recent years, with the continuous development and innovation of TCM techniques, acupuncture, as a safe and effective complementary therapy, has increasingly demonstrated its unique advantages in the treatment of systemic autoimmune diseases (12). In particular, it has provided novel insights and practical approaches for managing AS, a clinically intractable disease (13). Notably, the reason why these acupuncture methods can be effective in AS treatment lies in their adherence to the TCM principle of syndrome differentiation and treatment (14), combined with precise acupoint selection based on the individual disease conditions of AS patients. This ultimately achieves a multi-targeted and multi-pathway comprehensive regulatory effect: it not only regulates the body’s immune function and metabolic levels but also alleviates the core symptoms of patients through anti-inflammatory and analgesic effects (14, 15).

Given the scattered clinical evidence and lack of systematic reviews on acupuncture for AS, this study is necessary to summarize existing evidence via meta-analysis to clarify acupuncture’s efficacy and safety. This study systematically reviews current evidence to evaluate the effectiveness and safety of acupuncture in treating AS. It further explores the mechanisms of action of various acupuncture therapies, as well as existing limitations and potential areas for improvement, aiming to provide evidence for the acupuncture treatment of AS.

## Methods

This review was registered in PROSPERO (ID CRD42025633263) on January 7, 2025. At the study’s initiation, we predefined three primary outcomes (BASDAI, BASFI, ASDAS) and designated VAS, CRP, and ESR as secondary outcomes. The review was conducted and reported in accordance with the PRISMA 2020 guidelines.

### Data sources

In accordance with the Cochrane Handbook, we systematically searched PubMed, MEDLINE, Web of Science, and the Cochrane Central Register of Controlled Trials (CENTRAL), as well as the Chinese databases China National Knowledge Infrastructure (CNKI), Wanfang, and VIP, from their inception to December 25, 2024. For the English-language databases, searches were conducted using a combination of Medical Subject Headings (MeSH) terms and free-text terms related to ankylosing spondylitis and acupuncture interventions, applied to the title and abstract fields, with MeSH terms used where applicable. The search terms were then translated into Chinese and used to search the Chinese-language databases. All retrieved records were imported into Zotero for de-duplication.

### Study selection

During study selection, we did not exclude trials based on methodological quality (e.g., randomization, allocation concealment, blinding). We included (a) Study design: randomized controlled trials (RCTs); (b) Participants: patients diagnosed with ankylosing spondylitis (AS); (c) Experimental intervention: acupuncture therapies, including traditional needling, electroacupuncture, warm needling, fire needling, acupotomy, and related modalities; (d) Control intervention: sham acupuncture, conventional Western medicine, traditional Chinese medicine, or three-arm trials in which acupuncture could be treated as an independent comparison; (e) Outcomes: reporting at least one predefined outcome indicator. We excluded studies that: (a) were not RCTs; (b) did not target AS or only reported outcomes related to comorbidities; (c) evaluated traditional Chinese medicine therapies other than acupuncture or moxibustion; (d) did not provide outcome data on the efficacy of acupuncture for AS; (e) lacked accessible full-text data.

Primary outcome indicators:

- BASDAI (Bath Ankylosing Spondylitis Disease Activity Index).
- BASFI (Bath Ankylosing Spondylitis Functional Index).
- ASDAS (Ankylosing Spondylitis Disease Activity Score).

Secondary outcome indicators:

- VAS (Visual Analogue Scale).
- CRP (C-reactive protein).
- ESR (Erythrocyte Sedimentation Rate).

### Methodology quality assessment and data extraction

Two reviewers (JZ, SS) independently conducted literature screening. Any discrepancies arising at any screening stage were resolved through in-depth discussion with a third independent researcher (RB), followed by the extraction of data on: (a) author and publication year; (b) sample size; (c) details of interventions (acupuncture type, acupoints, treatment duration, single treatment duration) for treatment and control groups; (d) outcome measures; (e) adverse events. All information is recorded in Table 1.

**Table 1:** Baseline characteristics of included RCTs on acupuncture for ankylosing spondylitis.

First author (year)	Type of acupuncture	Sample sizes		Interventions		Acupuncture points selection	Single treatment duration	Treatment duration	Main outcomes	Adverse events
		T	C	T	C					
Wang Fei 2017 (27)	Acupuncture	36	36/36	Acupuncture+TCM	TCM/Western medicine	DU3, DU14, DU8, DU9, BL23, DU2, GB24	30 min	3 weeks	BASDAI, VAS, BASFI, CRP, ESR	Nausea, anorexia, fever, dermatitis, palpitations, dizziness, and abdominal pain
Chen Xin 2018 (50)	Electroacupuncture	54	54	Electroacupuncture	Conventional Western Medicine	C1-L5, BL23, DU14, BL11, BL18	30 min 2 Hz	13 weeks	VAS, CRP, ESR	n.r.
Thanakorn Theerakarunwong 2021 (26)	Du Mai Pai Zhen Method	31	30/32/30	Du Mai Pai Zhen Method+TCM	Acupuncture/Du Mai Pai Zhen Method/TCM	BL11, DU12, DU3, EX-B2	30 min	4 weeks	BASDAI, VAS, BASFI, CRP, ESR	Nausea, vomiting, belching
Zhuang Yu (38)	Warm Acupuncture	35	35	Warm Acupuncture	Western medicine	DU20, DU16, DU14, DU12, DU4, DU3, DU1	40 min	5 weeks of treatment over 10 weeks	BASDAI, BASFI, CRP, ESR	n.r.
Chen Yutao (19)	Silver needles	15	15/15	Acupuncture	TCM/Acupuncture+TCM	n.r.	n.r.	12 weeks	BASDAI	n.r.
Gao Lixin (42)	Fire Acupuncture	31	31	Western medicine+Fire Acupuncture	Western medicine	Du3, BL18, BL23, ST36, SP6	n.r.	12 weeks	BASDAI, BASFI, CRP, ESR	n.r.
Zheng Yimin (14)	Warm Acupuncture	35	35	Warm Acupuncture+Western medicine	Western medicine	SP16, SP15, SP14, SP9, GB34, ST36, ST37, ST40	60 min	4 weeks	BASDAI, BASFI, CRP, ESR, ASDAS	Abdominal distension, diarrhea
Wang Yingjie 2017 (40)	Floating needle	40	40	Floating needle	Western medicine	Lower back, buttocks	30 min	24 weeks	BASDAI, BASFI	n.r.
Guo Chunliang 2024 (47)	Exercise needles	47	47	Exercise needles+Western medicine+Tuina	Western medicine+Tuina	KI6, BL62	n.r.	32 weeks	BASDAI, VAS, BASFI, CRP, ESR	n.r.
Wu Jia 2023 (48)	Exercise needles	39	39	Exercise needles+Western medicine	Western medicine	KI6, BL62	20 min	3 weeks	BASDAI, BASFI, CRP	Vomiting, abnormal liver function
Liu Biyan 2019 (23)	Acupuncture	40	40	Western medicine+Acupuncture	Acupuncture	EX-B2, BL23, BL25, BL54, GB30, BL40	30 min	8 weeks	BASDAI, BASFI, CRP	n.r.
Gao Taozhen 2019 (29)	Warm Acupuncture	20	20	Western medicine+Warm Acupuncture	Western medicine	EX-B2	30 min	4 weeks	BASFI, CRP, ESR	n.r.
Luo Yuxuan 2024 (24)	Acupuncture	30	30	Western medicine+Acupuncture	Western medicine	DU20, DU14, HT7, BL23, BL18, DU3, GB34, SP6	30 min	4 weeks	BASDAI, BASFI, CRP, ESR	n.r.
Huang Yongjie 2016 (22)	Acupuncture	43	43	Western medicine+Acupuncture	Western medicine	SP6, BL40, LI15, LI4, LI11, LI10	30 min	5 weeks	BASDAI, BASFI, CRP, ESR	n.r.
Dai Chaoran 2020 (51)	Electroacupuncture	29	29	Electroacupuncture+Western medicine	Western medicine	BL10, BL11, DU14, BL27, BL32, SJ5, GB34, EX-B2	30 min, 1–2 mA 2 Hz	2 weeks	BASDAI, VAS, BASFI, CRP, ESR	n.r.
Yang Lihua 2020 (34)	Warm Acupuncture	34	34	Warm Acupuncture	Western medicine	DU14, GB20, BL11, BL23	30 min	8 weeks	VAS, CRP, ESR	n.r.
Wang Junren 2013 (33)	Warm Acupuncture	30	30	Acupuncture	Western medicine	EX-B2, DU2, DU14, DU1, DU5, BL40, BL18	30 min	12 weeks	BASDAI, BASFI, CRP, ESR, VAS	n.r.
Zhou Shaohui 2011 (37)	Warm Acupuncture	28	28	Warm Acupuncture+Western medicine	Western medicine	DU14, BL11, BL18, BL23, EX-HN14	30 min	3 weeks	BASDAI, BASFI, CRP, ESR	n.r.
Wang Zhengrong 2022 (44)	Needle knives	40	40	Needle knives+Western medicine	Western medicine	Lumbosacral, Abdominal regions	n.r.	8 weeks	BASDAI, BASFI, CRP, ESR	n.r.
Lin Bo 2023 (31)	Warm Acupuncture	40	40	Warm Acupuncture+Herbal Fumigation	Herbal Fumigation	DU20, DU16, DU14, DU12, DU4, DU3, DU1	40 min	12 weeks	BASDAI, BASFI, CRP, ESR, VAS	skin allergies, subcutaneous hematoma, nausea and vomiting
You Yuquan 2020 (46)	Needle knives	30	30/30	Needle knives	Needle knives+Western medicine/Western medicine	sacroiliac joint	n.r.	24 weeks	BASDAI, BASFI, CRP, ESR	n.r.
Huang Wenchao 2024 (30)	Warm Acupuncture	26	26	Western medicine+Warm Acupuncture	Western medicine	SP16, SP15, GB34, SP9, ST36, ST40, ST37	60 min	n.r.	BASFI	n.r.
Ye Lei 2022 (35)	Warm Acupuncture	30	30	Western medicine+Warm Acupuncture	Western medicine	BL23, BL26, BL28, GB29, GB30	30 min	5 weeks	BASDAI, BASFI, VAS	n.r.
Zhao Mingyang 2023 (28)	Acupuncture	30	30	Western medicine+Acupuncture	Western medicine	TSBh1-8910, RFh1-6, RFh2-6, DXh2-6811, DNsz, DWSg, DZBh1-5	30 min	4 weeks	BASDAI, VAS, CRP, ESR	C: Gastrointestinal upset
Gao Shouyuan 2019 (20)	Acupuncture for Tonifying the Kidney	32	32	Acupuncture	Western medicine	DU14, BL18, BL20, DU4, BL23, ST36, SP6, KI3	30 min	6 weeks	BASDAI, BASFI, VAS	n.r.
Wang Dongzhi 2020 (15)	Warm Acupuncture	30	30	Moxibustion supervision+Warm Acupuncture	Moxibustion supervision	n.r.	60 min	22 weeks	CRP, ESR	n.r.
Luo Xiaoguang 2022 (32)	Warm Acupuncture	40	40	Proprietary chinese medicines +Warm Acupuncture	Proprietary chinese medicines	BL22, BL23, RN6, BL25, RN4, BL29, BL30	n.r.	8 weeks	BASDAI, BASFI, CRP, ESR, VAS	n.r.
Li Xinxian 2015 (52)	Bee needles	30	30	Bee needles+Electroacupuncture	Electroacupuncture	EX-B2, BL28, BL23, DU16, DU14, BL13, BL67, BL25, DU3	n.r.	4 weeks	BASDAI, ASDAS, CRP, ESR, VAS	n.r.
Zeng Yufen 2017 (41)	Floating needle	48	50	Floating needle+TCM	Western medicine+TCM	MTrP	10 min	3 weeks	VAS	n.r.
Zeng Wenbi 2018 (43)	Fire needle	23	23	Western medicine+Fire needle	Western medicine	BL13, BL14, BL15, BL18, BL19, BL20, BL22, BL.23, BL25, BL28	5 min	4 weeks	VAS	n.r.
Shi Wencai 2020 (25)	Acupuncture	30	30/30	Western medicine+TCM + Acupuncture	Western medicine+TCM/Western medicine	BL11, DU14, BL18, BL23	30 min	8 weeks	BASDAI, BASFI, CRP, ESR	n.r.
Wang Zhaoyi 2022 (45)	The meridian puncture method	49	49	The meridian puncture method+Western medicine	Western medicine	Foot sun meridian tendon junction lesions	30 min	8 weeks	CRP, ESR	n.r.
Hou Haikun 2021 (21)	Wen yang Tong Du needlezy	49	49	Wen yang Tong Du needle+Inner heat needle	Inner heat needle	KI3, KI1, SP9, GB34, DU20, GB21	30 min	4 weeks	BASFI, CRP, ESR, VAS	n.r.
Zhang Le 2017 (36)	Warm acupuncture	30	31	Western medicine+Warm acupuncture	Western medicine	BL23, DU4, BL40, GB30, DU3	n.r.	2 weeks	ESR	n.r.
Lu Zhishu 2024 (39)	Floating needle	34	34	Western medicine+Floating needle	Western medicine	MTrP	240 min	8 weeks	BASDAI, BASFI	Subcutaneous hematoma, skin rash, dyspepsia

T, treatment group; C, control group; n.r., not reported; CRP, C-reactive protein; ESR, Erythrocyte Sedimentation Rate; VAS, Visual Analogue Scale; BASDAI, Bath Ankylosing Spondylitis Disease Activity Index; BASFI, Bath Ankylosing Spondylitis Functional Index; ASDAS, ASAS Spondyloarthritis Disease Activity Score; TCM, Traditional Chinese Medicine.

We used the Cochrane risk-of-bias tool to assess the risk of: (a) bias in random sequence generation; (b) bias in allocation concealment; (c) performance bias (blinding of participants and personnel); (d) detection bias (blinding of outcome assessment); (e) attrition bias (incomplete outcome data); (f) reporting bias (selective reporting); and (g) other biases, and then determine whether the type of risk is “high-risk” (H), “low-risk” (L), or “unclear-risk” (U) (16). Any disagreements were resolved by consensus or through consultation with a third reviewer (RB).

### Data analysis

If essential data were missing, we attempted to contact study authors for clarification. We used Review Manager (RevMan 5.4) for meta-analysis. For continuous outcomes, we pooled data as mean differences (MD) with 95% confidence intervals (CI); for dichotomous outcomes, we used relative risk (RR) with 95% CI. Heterogeneity was assessed with the I^2^ statistic (17). If there is significant heterogeneity among the included trials, a random-effects model will be used for pooled meta-analysis. If heterogeneity is high, subgroup analysis or sensitivity analysis will be applied to manage the data.

- An I^2^ < 25% was considered low heterogeneity, in which case a fixed-effect model might be appropriate, and pooled results are relatively reliable.
- I^2^ of 25–50% indicated moderate heterogeneity, and we explored potential sources of heterogeneity (e.g., subgroup analyses).
- I^2^ ≥ 50% was considered high heterogeneity. In such cases, we applied a random-effects model and planned subgroup or sensitivity analyses. We also intended to explore the reasons for heterogeneity in the Discussion field (18).

## Results

### Retrieval results and document information

**Figure 1:**
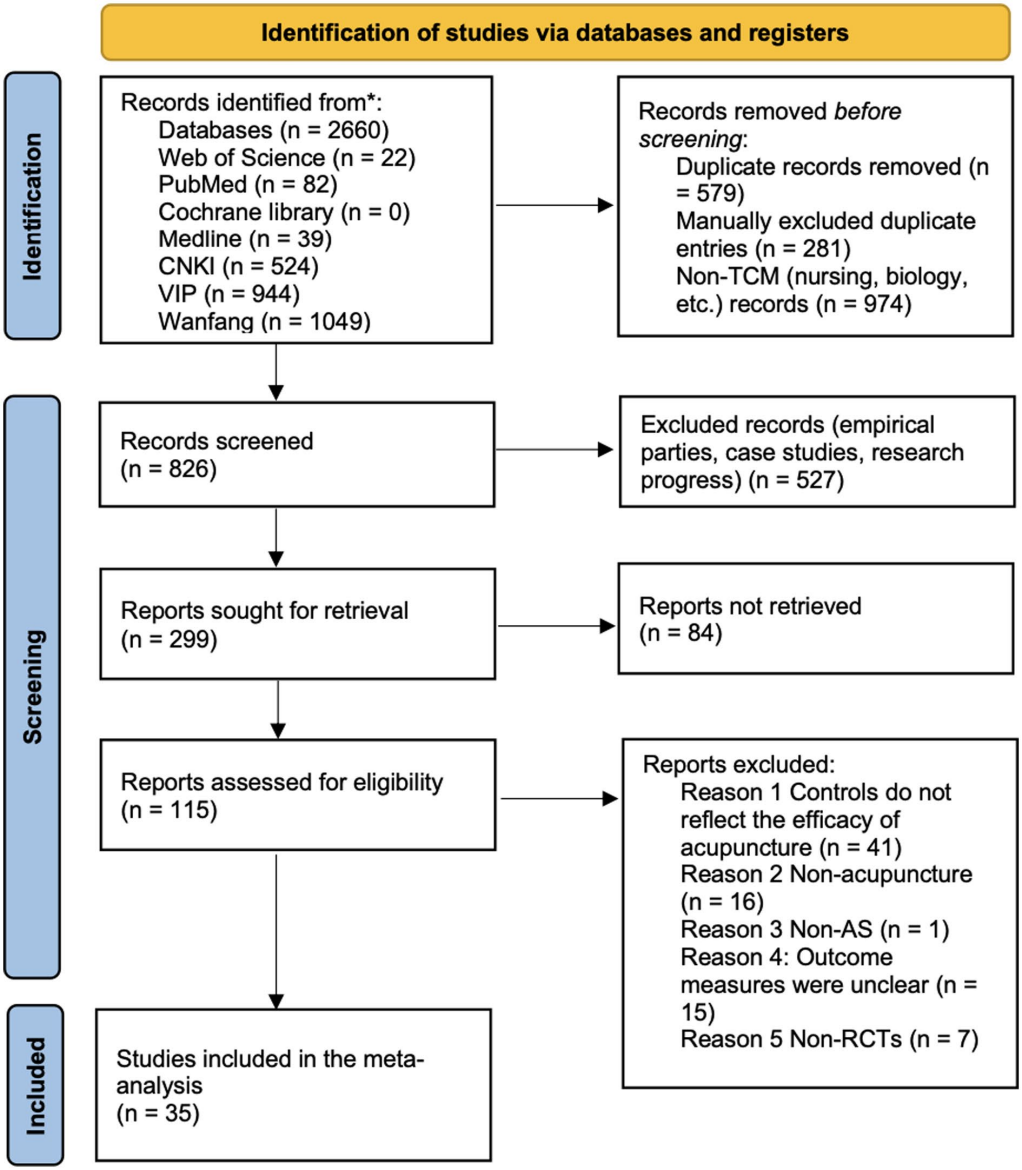
Flowchart of the literature review and selection process.

Initially, a total of 2,660 records were identified. After removing 860 duplicates (579 by software and 281 manually) and 1,465 irrelevant or ineligible records (non-RCTs, unrelated topics, etc.), 35 RCTs remained for meta-analysis. The specific information contained in these studies was summarized in Figure 1. Altogether, these studies included 2,591 AS patients, with individual sample sizes ranging from 40 to 123. Among the included studies, ten studies evaluated traditional manual acupuncture (19–28), twelve studies evaluated warm acupuncture (14, 15, 29–38), three studies evaluated floating acupuncture (39–41), two studies used fire acupuncture (42, 43), two studies used acupotomy (needle-knife) (44–46), two studies used exercise (kinetic) acupuncture (47, 48), two studies used electroacupuncture (49–51), one study used meridian needling (44, 45), and one study used bee venom acupuncture (52).

Among the included studies, five studies were three-arm trials, with TCM, Western medicine, and acupuncture used as mutual controls (19, 25–27, 46); three studies set acupuncture controls based on acupuncture intervention (21, 44, 45, 52); one study used TCM alone (41), and one study used Chinese patent medicine alone (32); one study added acupuncture control based on moxibustion (15), one study used TCM fumigation and washing therapy as the control (31), and one study set a control based on “tuina and Western medicine” (47); the remaining studies all used Western medicine as the control (e.g., NSAIDs or DMARDs), except for the studies using acupotomy. The common acupoints included BL23, BL25, DU14, EX-B2, DU4, and DU3, involving primarily the Governor Vessel (Du) and Bladder meridians, along with points from the Spleen and Gallbladder meridians.

Seven studies reported various adverse reactions (14, 26–28, 31, 39, 48), such as abdominal bloating, diarrhea, nausea, anorexia, fever, dermatitis, palpitations, dizziness, and abdominal pain. In addition, the detailed characteristics of each included study are presented in Table 1. Most reported adverse events occurred in participants receiving concomitant pharmacologic therapy and were unlikely to be directly caused by acupuncture. No trial reported a serious acupuncture-related adverse event, and the events attributable specifically to acupuncture (e.g., transient dizziness or localized discomfort) were mild and self-limiting. In terms of treatment delivery, acupuncture sessions typically lasted 30 min, with a small number of studies extending the duration to 40 min or 1 h (14, 15, 38, 39). In particular, the acupuncture time was not mentioned for the acupotomy therapy. Treatment duration ranged from 2 weeks to 32 weeks across studies.

### Risk of bias

**Figure 2:**
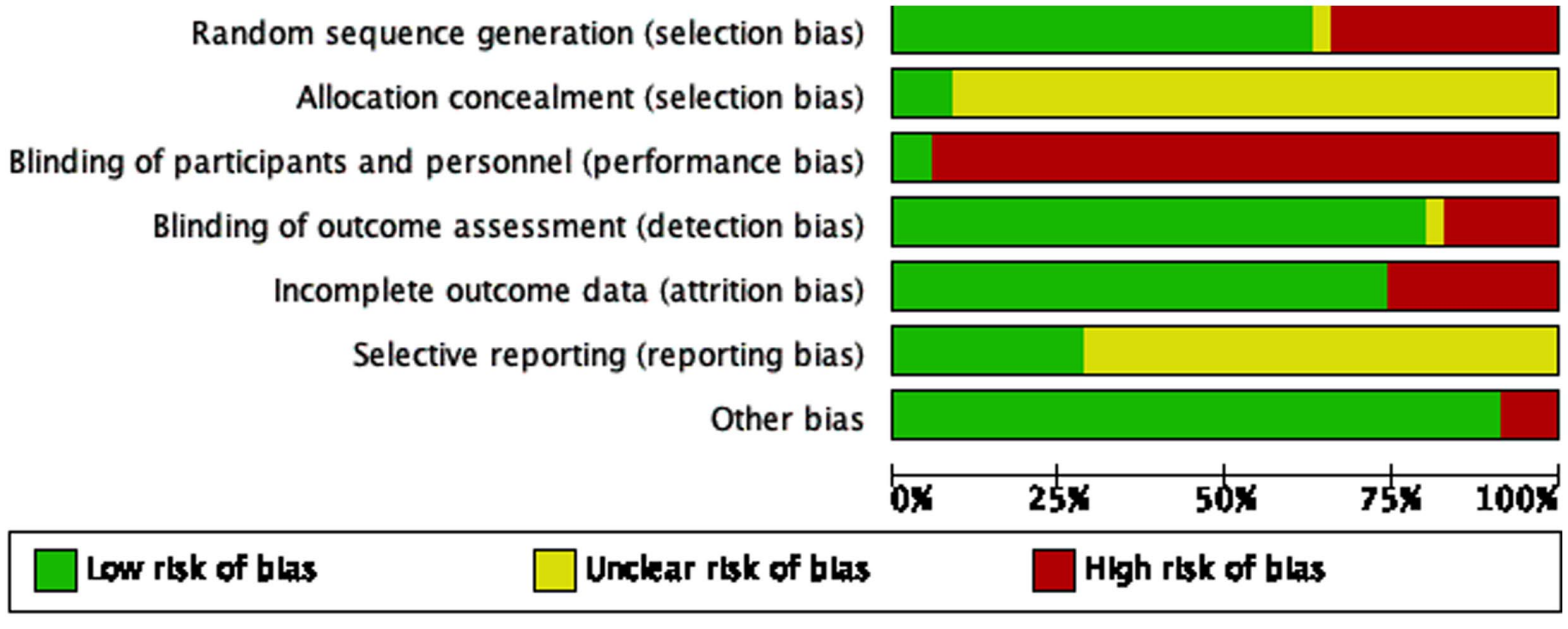
Assessment of cochrane risk of bias presented as percentages across all included studies.

**Figure 3:**
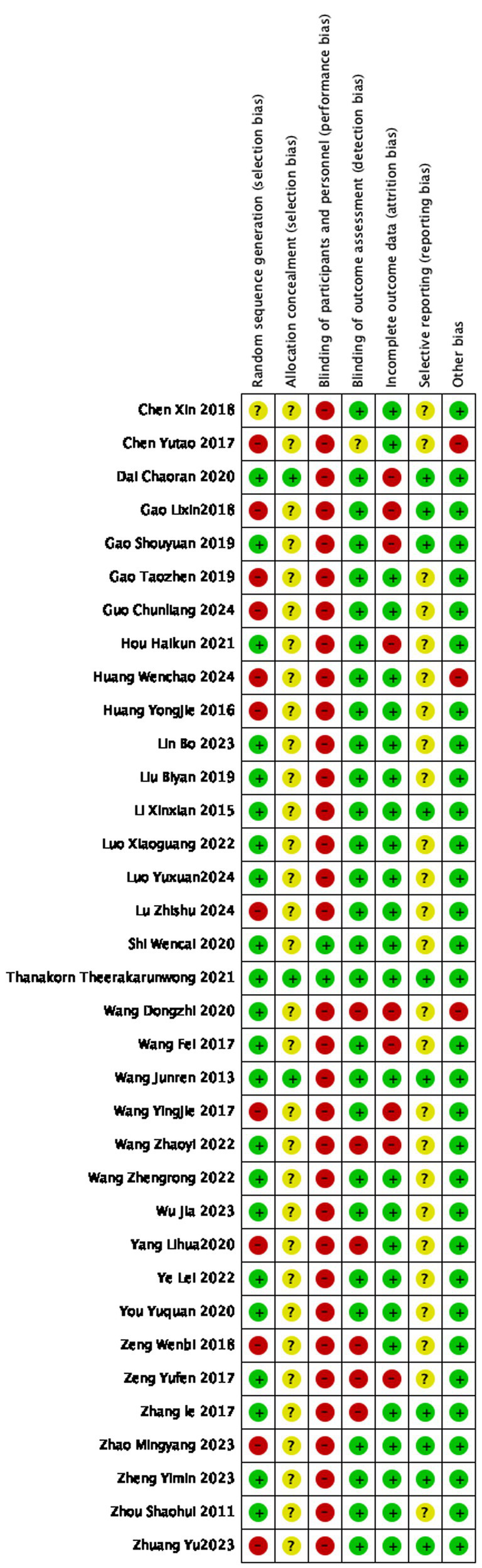
Cochrane risk of bias summary for each included study.

The risk of bias graph is presented in Figure 2, and the risk of bias summary is shown in Figure 3. Additionally, the summary of the risk of bias is documented in Table 2.

**Table 2:** Risk of bias for the 35 included studies using the Cochrane risk of bias tool.

Study (first author, year)	Random sequence generation	Allocation concealment	Blinding of participants and personnel	Blinding of outcome assessors	Incomplete outcome	Selective reporting	Other sources of bias
Wang Fei 2017 (27)	L	U	H	L	H	U	L
Chen Xin 2018 (50)	U	U	H	L	L	U	L
Thanakorn Theerakarunwong 2021 (26)	L	L	L	L	L	L	L
Zhuang Yu 2023 (38)	H	U	H	L	L	L	L
Chen Yutao 2017 (19)	H	U	H	U	L	U	H
Gao Lixin 2018 (42)	H	U	H	L	H	L	L
Zheng Yimin 2023 (14)	L	U	H	L	L	L	L
Wang Yingjie 2017 (40)	H	U	H	L	H	U	L
Guo Chunliang 2024 (47)	H	U	H	L	L	U	L
Wu Jia 2023 (48)	L	U	H	L	L	U	L
Liu Biyan 2019 (23)	L	U	H	L	L	U	L
Gao Taozhen 2019 (20)	H	U	H	L	L	U	L
Luo Yuxuan 2024 (24)	L	U	H	L	L	U	L
Huang Yongjie 2016 (22)	H	U	H	L	L	U	L
Dai Chaoran 2020 (51)	L	L	H	L	H	L	L
Yang Lihua 2020 (34)	H	U	H	H	L	U	L
Wang Junren 2013 (33)	L	L	H	L	L	L	L
Zhou Shaohui 2011 (37)	L	U	H	L	L	U	L
Wang Zhengrong 2022 (44)	L	U	H	L	L	U	L
Lin Bo 2023 (31)	L	U	H	L	L	U	L
You Yuquan 2020 (46)	L	U	H	L	L	U	L
Huang Wenchao 2024 (30)	H	U	H	L	L	U	H
Ye Lei 2022 (35)	L	U	H	L	L	U	L
Zhao Mingyang 2023 (28)	H	U	H	L	L	L	L
Gao Shouyuan 2019 (29)	L	U	H	L	H	L	L
Wang Dongzhi 2020 (15)	L	U	H	H	H	U	H
Luo Xiaoguang 2022 (32)	L	U	H	L	L	U	L
Li Xinxian 2015 (52)	L	U	H	L	L	L	L
Zeng Yufen 2017 (41)	L	U	H	H	H	U	L
Zeng Wenbi 2018 (43)	H	U	H	H	L	U	L
Shi Wencai 2020 (25)	L	U	L	L	L	U	L
Wang Zhaoyi 2022 (45)	L	U	H	H	H	U	L
Hou Haikun 2021 (21)	L	U	H	L	H	U	L
Zhang Le 2017 (36)	L	U	H	H	L	L	L
Lu Zhishu 2024 (39)	H	U	H	L	L	U	L

“L”, a low risk of bias; “U”, the risk of bias is uncertain; “H”, a high risk of bias.

### Selection bias

Among the 35 included studies, one study did not describe the randomization process (49, 50). Among the remaining studies, twelve studies did not used random number tables for randomization (19, 22, 28–30, 34, 38–40, 42, 43, 47) were judged to be at high risk of bias.

Only three studies described the method of random allocation; these studies were assessed as having low risk of bias because they achieved allocation concealment using sealed envelopes (26, 33, 51), while the other thirty-two studies either did not describe their allocation methods or performed random allocation based on admission order. Overall, most studies had a high risk of bias in terms of random sequence generation and allocation concealment.

### Performance bias

Among the included RCTs, thirty-three studies were rated as having a high risk of performance bias because they did not provide sufficient information to demonstrate that blinding of participants or study personnel was implemented. In contrast, only two studies (25, 26) explicitly reported a plausible double-blinding procedure and were therefore not classified as high risk.

### Detection bias

Among the 35 included studies, six studies (15, 34, 36, 41, 43–45) were considered at high risk of detection bias due to their reliance on simplistic or subjective outcome measures, such as single self-reported indicators, which may compromise the reliability of effect estimates. Furthermore, one study had a very small sample and provided insufficient detail regarding outcome assessment procedures; as a result, its detection bias was rated as “unclear” (19). These factors should be taken into account when interpreting the pooled results.

### Attrition bias

Nine studies reported patient drop-outs (due to adverse events), so attrition bias was high in these (15, 20, 21, 27, 40–42, 44, 45, 51). The other studies reported no loss to follow-up, indicating low attrition bias. The remaining twenty-six studies had no data loss and were judged as low risk.

### Reporting bias

Twenty-five studies did not provide information on trial registration or protocols, hence were judged “unclear” for reporting bias. Only ten studies that reported all expected outcomes were considered at low risk (14, 20, 26, 28, 33, 36, 38, 42, 51, 52). Registration information was not reported in most Chinese and English studies; therefore, the risk of reporting bias was mostly assessed as “unclear-risk.”

### Other biases

One study was assessed as high risk of bias due to insufficiently disclosed information (30), and two studies were judged to have high risk of bias because details such as acupuncture points were not clearly specified (15, 19). No other sources of bias were apparent.

### Primary outcome indicators

#### BASDAI score

**Figure 4:**
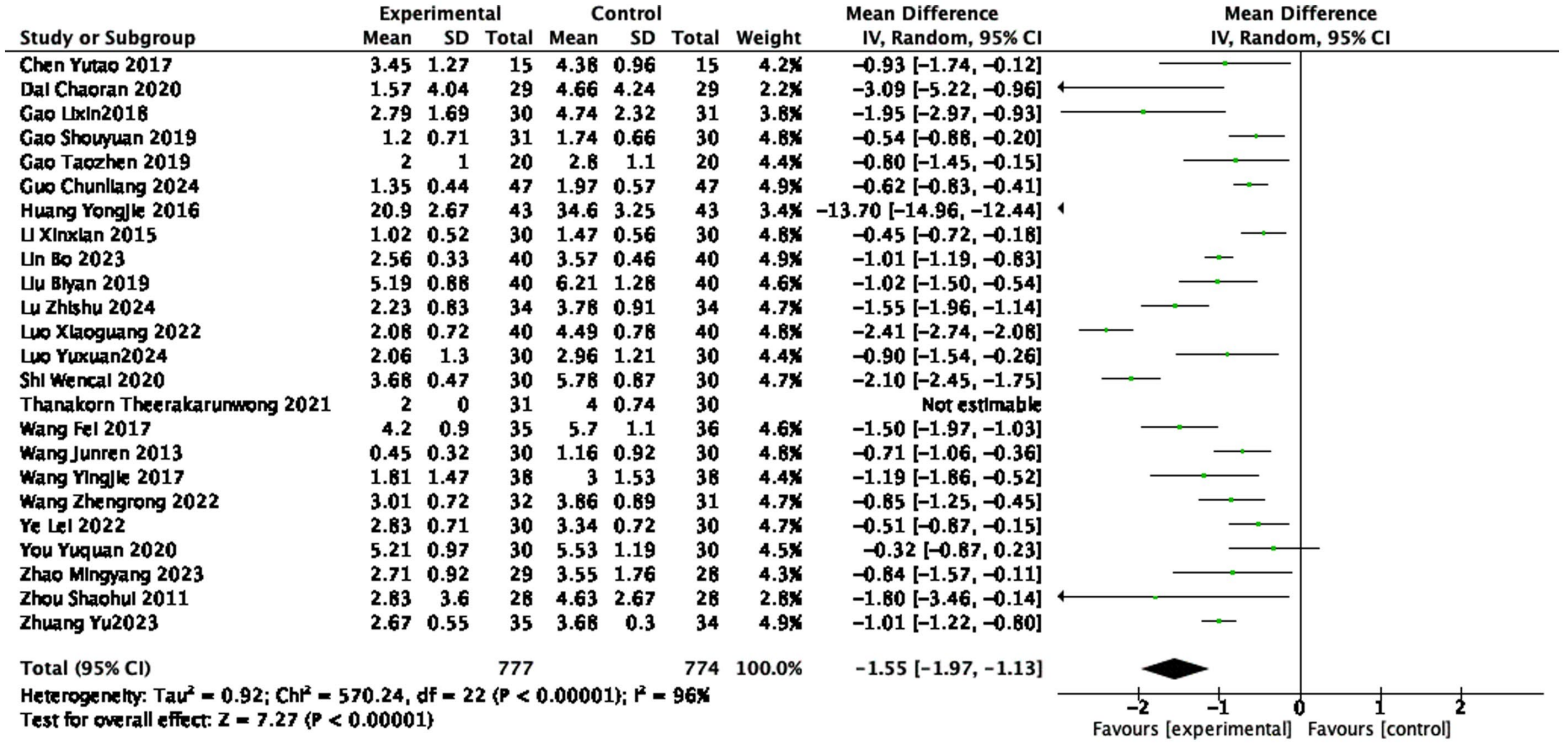
Forest plot of BASDAI score.

Twenty-four studies (19, 20, 22–29, 31–33, 35, 37–40, 42, 44–47, 51, 52) reported the BASDAI scores after treatment, involving 777 patients in the treatment group and 774 patients in the control group. Considerable heterogeneity was observed (I^2^ = 96%), so a random-effects model was used. Despite the heterogeneity, all studies showed a direction of effect favoring acupuncture. The BASDAI score in the treatment group was lower than that in the control group (1,551 participants, MD = −1.55, 95% CI: −1.97 to −1.13, *p* < 0.0001), with a statistically significant difference (see Figure 4).

#### BASFI score

**Figure 5:**
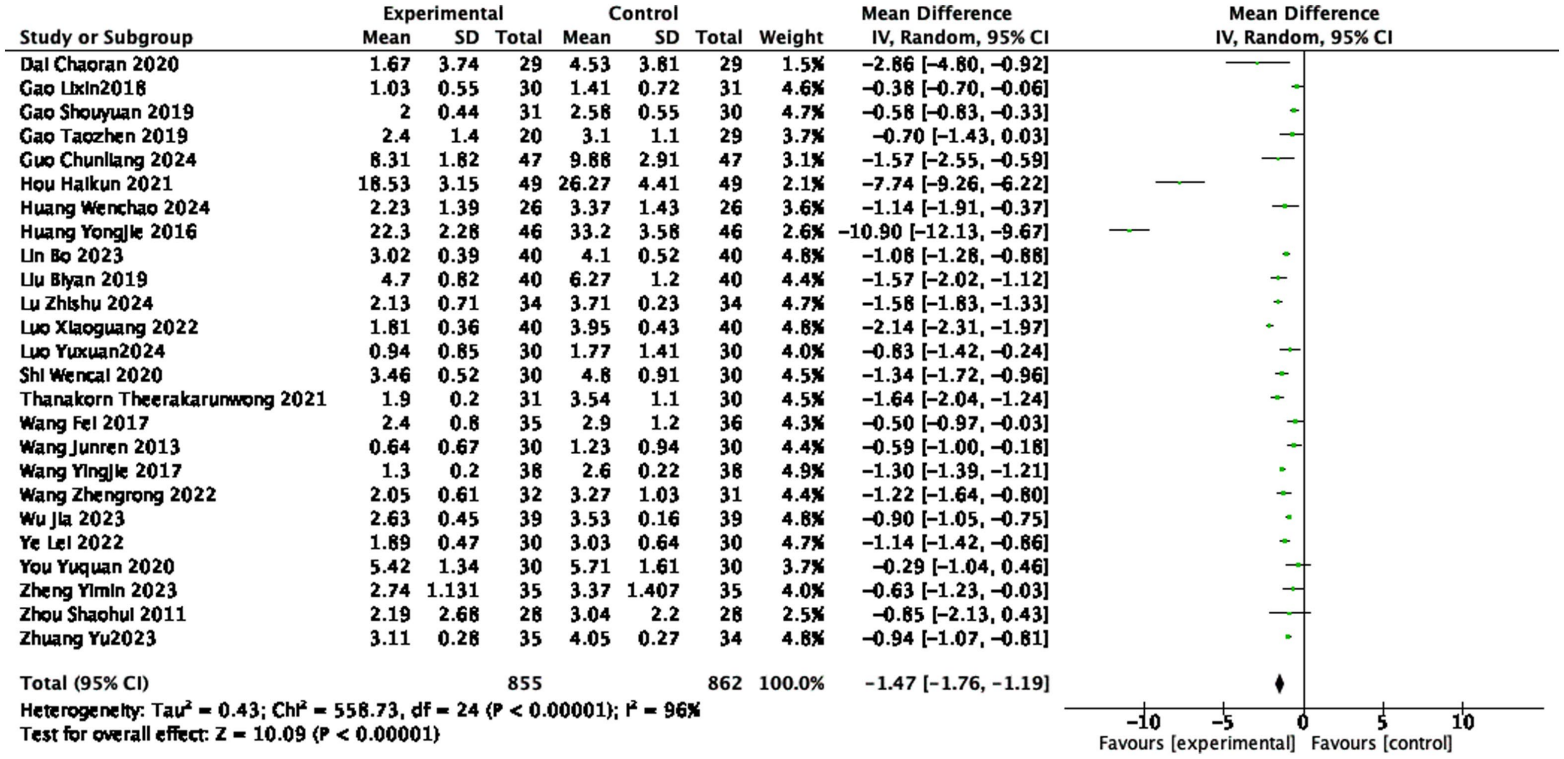
Forest plot of BASFI score.

Twenty-five studies (14, 20–27, 29–33, 35, 37–40, 42, 44–48, 51) reported the BASFI scores after treatment, involving 855 patients in the treatment group and 862 patients in the control group. The heterogeneity is very high (I^2^ = 96%, random effect model), but the overall results show that acupuncture and moxibustion can significantly reduce the BASFI score. The BASFI score in the treatment group was lower than that in the control group (1,717 participants, MD = −1.47, 95% CI: −1.76 to −1.19, *p* < 0.0001), with a statistically significant difference (see Figure 5).

#### ASDAS

**Figure 6:**
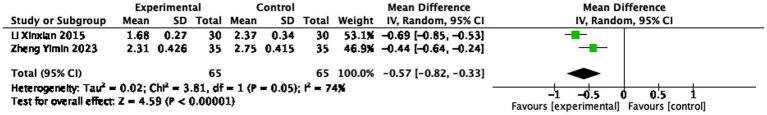
Forest plot of ASDAS score.

Two studies (14, 52) reported the ASDAS after treatment, involving 65 patients in the treatment group and 65 patients in the control group. The heterogeneity of the analysis was I^2^ = 74%, indicating statistically significant differences among the studies. The ASDAS in the treatment group was lower than that in the control group (130 participants, MD = −0.57, 95% CI: −0.82 to −0.33, *p* < 0.0001), with a statistically significant difference (see Figure 6).

### Secondary outcome indicators

#### VAS score

**Figure 7:**
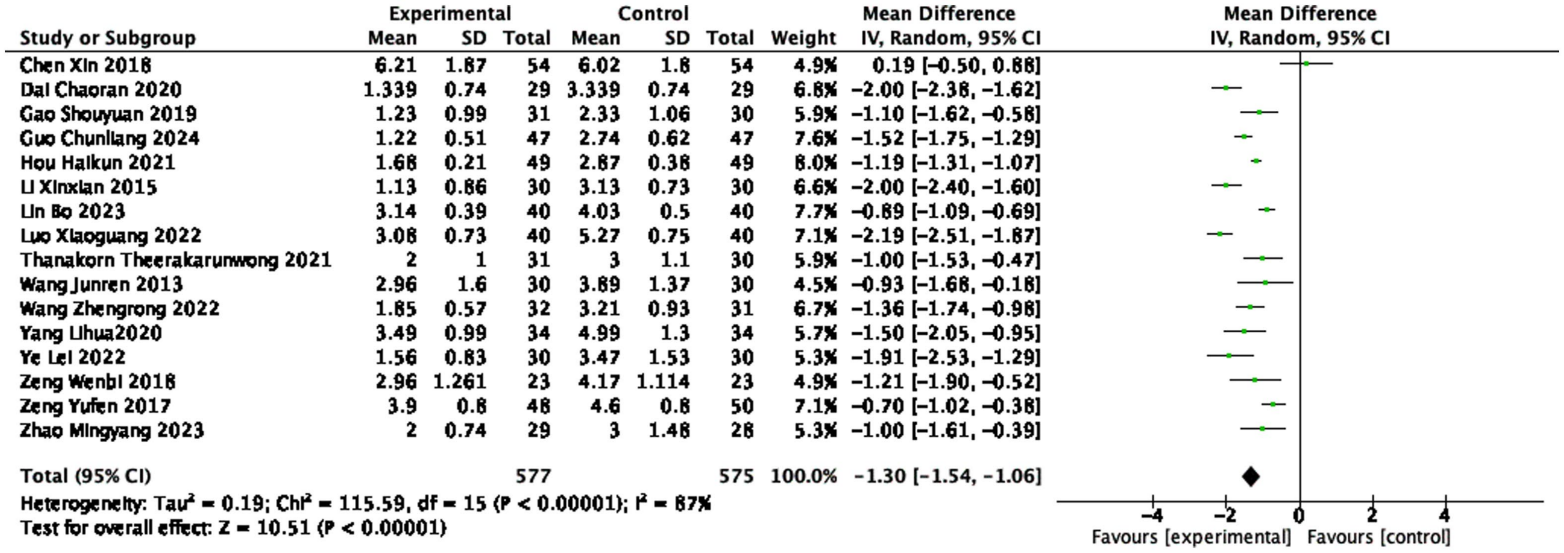
Forest plot of VAS score.

Sixteen studies (20, 21, 26, 28, 31–35, 41, 43–45, 47, 49–52) reported the VAS scores after treatment, involving 577 patients in the treatment group and 575 patients in the control group. Heterogeneity was substantial (I^2^ = 87%, random-effects model), indicating statistically significant differences among the studies. The VAS score in the treatment group was lower than that in the control group (1,152 participants, MD = −1.30, 95% CI: −1.54 to −1.06, *p* < 0.0001), with a statistically significant difference (see Figure 7).

#### CRP level

**Figure 8:**
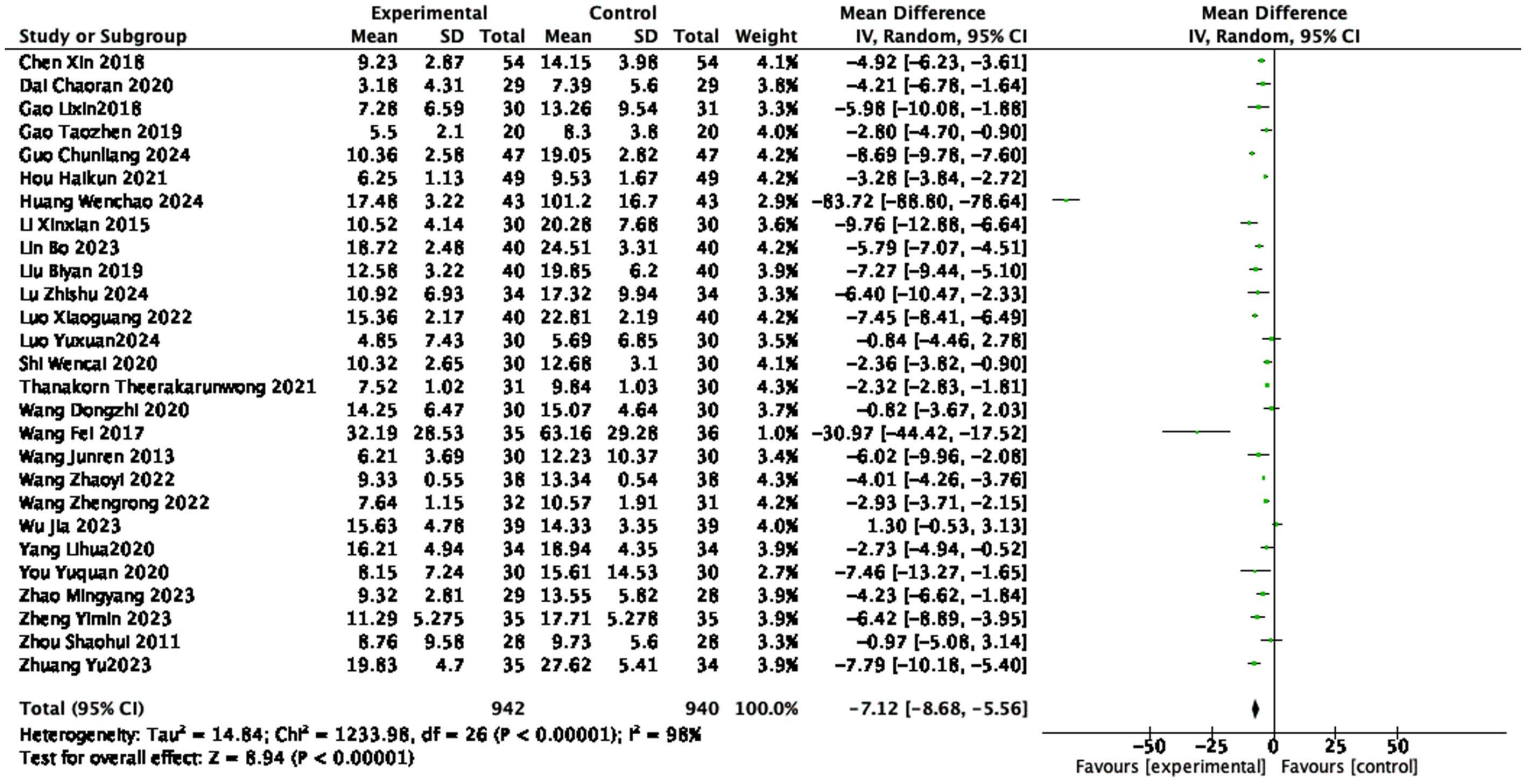
Forest plot of CRP score.

Twenty-seven studies (14, 15, 21, 23–34, 37–39, 42, 44–52) reported the CRP level after treatment, involving 942 patients in the treatment group and 940 patients in the control group. Extremely high heterogeneity (I^2^ = 98%), using a random effects model, indicating statistically significant differences among the studies. The CRP level in the treatment group was lower than that in the control group (1882 participants, MD = −7.12 mg/L, 95% CI: −8.68 to −5.56, *p* < 0.0001, see Figure 8), with a statistically significant difference.

#### ESR level

**Figure 9:**
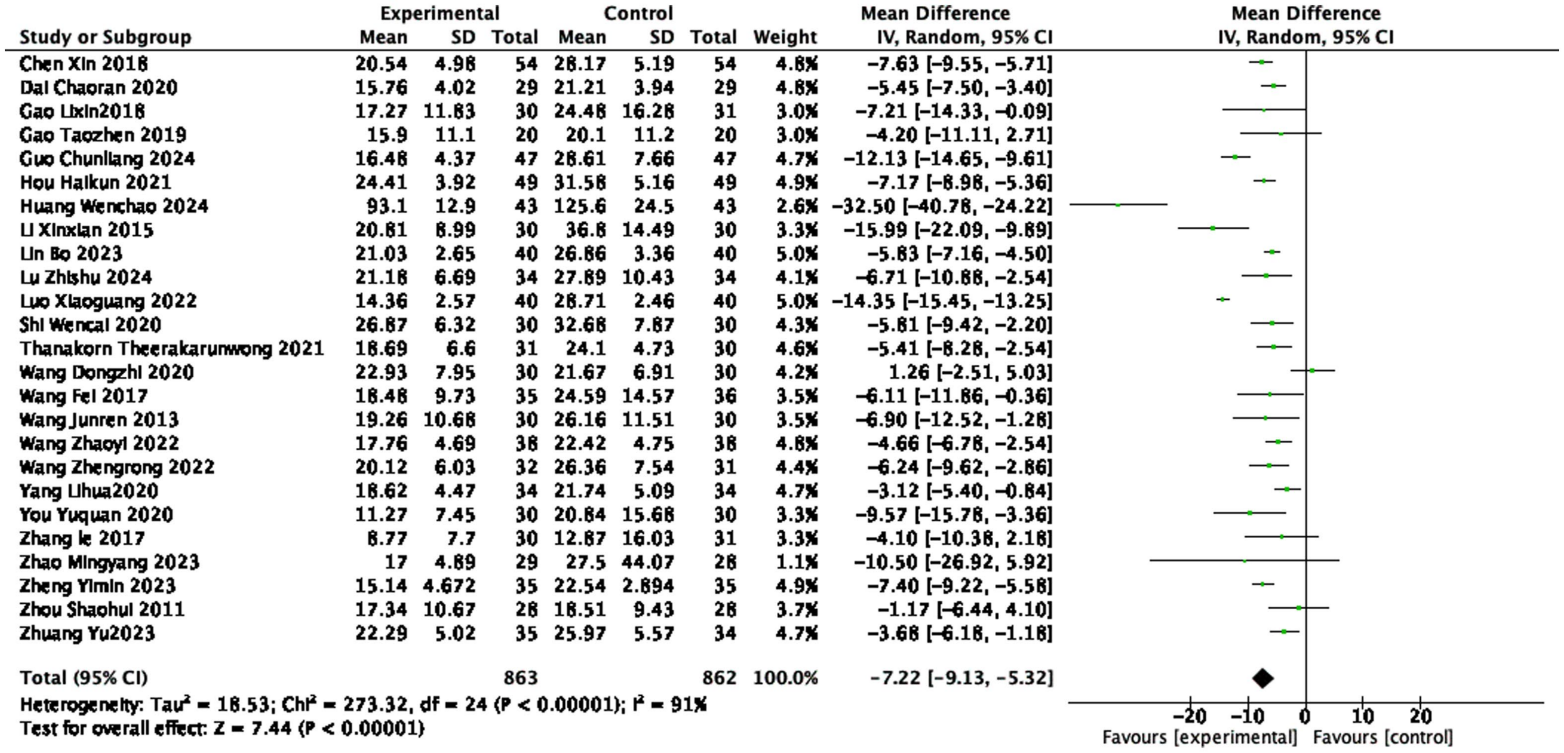
Forest plot of ESR score.

Twenty-five studies (14, 15, 21, 25–34, 36–39, 42, 44–47, 49–52) reported the ESR level after treatment, involving 863 patients in the treatment group and 862 patients in the control group. The heterogeneity was very high (I^2^ = 91%, random effect). Indicating statistically significant differences among the studies. The ESR level in the treatment group was lower than that in the control group (1725 participants, MD = −7.22 mm/h, 95% CI: −9.13 to −5.32, *p* < 0.0001, see Figure 9), with a statistically significant difference.

### Subgroup analysis

In this study, CRP was selected as the outcome for subgroup analysis because it was reported by the most studies, providing the largest sample size for analysis. Subgroup analyses were performed by different acupuncture types, each treatment duration, treatment frequency, and total course, with results detailed in Table 3. We found that significant differences in CRP results were observed across different acupuncture methods, intervention durations, and treatment cycles (detailed forest plots are available in Supplementary material 1).

**Table 3:** Results of subgroup analysis on the improvement of AS in patients with acupuncture intervention.

Term	Category	Included studies	Results of heterogeneity assessment		Effect model	Meta-analysis results		
			I^2^	*P*		MD	95%CI	*P*
Intervention form	Acupuncture	7	86%	*p* < 0.001	Random	−3.65	−4.97 to −2.32	*P* < 0.001
	Warm acupuncture	10	99%	*P* < 0.001	Random	−12.16	−18.77 to −5.56	*p* < 0.001
	Needle-knife therapy	2	56%	*p* = 0.13	Random	−4.24	−8.27 to −0.21	*p* = 0.04
	Fire acupuncture	1	n.r.	n.r.	Random	n.r.	n.r.	n.r.
	Floating needle	1	n.r.	n.r.	Random	n.r.	n.r.	n.r.
	Exercise-acupuncture	2	99%	*P* < 0.001	Random	−3.72	−13.51 to 6.07	*p* = 0.46
	Electroacupuncture	2	0%	*p* = 0.63	Random	−4.77	−5.94 to −3.61	*P* < 0.001
	Bee acupuncture	1	n.r.	n.r.	Random	n.r.	n.r.	n.r.
	Meridian needling	1	n.r.	n.r.	Random	n.r.	n.r.	n.r.
Single treatment duration	Less 30 min	1	n.r.	n.r.	Random	n.r.	n.r.	n.r.
	30 min	14	83%	*P* < 0.001	Random	−3.66	−4.44 to −2.88	*p* < 0.001
	More 30 min	6	99%	*p* = 0.009	Random	−18.32	−32.08 to −4.55	*p* < 0.001
Treatment duration	Less than 5 weeks	11	88%	*P* < 0.001	Random	−3.94	−5.27 to −2.60	*p* < 0.001
	5 weeks-10 weeks	8	90%	*p* < 0.001	Random	−4.67	−5.99 to −3.35	*P* < 0.001
	More than 10 weeks	6	87%	*P* < 0.001	Random	−5.59	−7.73 to −3.45	*p* < 0.001

n.r., not reported.

### Sensitivity analysis

Two sensitivity analyses were conducted to evaluate the robustness of the pooled results. First, a sensitivity analysis restricted to high-quality studies could not be performed because only one study was rated as having a low overall risk of bias (21). Second, the leave-one-out analysis showed that the pooled effect estimates and heterogeneity remained stable after sequential exclusion of each study, indicating that no single study exerted a disproportionate influence on the overall findings or served as the primary source of heterogeneity.

## Discussion

This study conducted a meta-analysis of 35 RCTs to clarify the core efficacy of acupuncture for ankylosing spondylitis (AS). Compared with conventional treatment alone, the acupuncture group demonstrated significantly greater improvements in disease activity (assessed via BASDAI and ASDAS), functional status (BASFI), and pain intensity (VAS score), alongside a marked reduction in inflammatory markers (ESR and CRP). Baseline levels of these markers were generally comparable between groups, reducing—but not eliminating—the possibility that post-treatment differences were influenced by initial imbalance. These findings provide quantitative evidence supporting acupuncture as a beneficial intervention for AS.

However, high heterogeneity was observed across all outcome measures. Further analysis identified the key potential contributors: inadequate control of confounding factors, including variations in acupuncture modalities, intervention durations, and assessment tools, which precluded the identification of core variables influencing outcomes. Although heterogeneity can be explored through a stepwise subgrouping approach, which involves holding two variables constant (e.g., assessment tool and intervention duration) while varying the third (e.g., needling method) to observe changes in I^2^, this strategy requires an adequate number of studies. Specifically, each resulting subgroup must contain at least two studies to allow meaningful comparison; otherwise, the findings cannot be considered robust.

Building on the above findings regarding efficacy and heterogeneity, we further explored the mechanisms underlying acupuncture’s symptom-alleviating effects in AS. From the perspective of shared mechanisms, existing studies have validated acupuncture’s effectiveness across multiple dimensions: (a) At the neural repair level, acupuncture along the Governor Vessel (Du Meridian) can promote the repair and regeneration of damaged neural tissue (53). For example, in an animal model of spinal cord injury, this approach upregulated the expression of microtubule-associated protein 2 (MAP-2) and neurofilament-light chain (NF-L), suggesting potential for improving neurofunction-related symptoms in AS patients (26); (b) At the immune regulation and bone protection level, acupuncture modulates the immunoinflammatory state of AS patients by reducing ESR and proinflammatory cytokine levels while increasing the expression of complement C3 and immunoglobulin A (IgA). Additionally, studies have observed that acupuncture may downregulate human leukocyte antigen-B27 (HLA-B27) expression to slow disease progression, promote central endorphin release for pain relief, and reduce levels of bone turnover enzymes (e.g., creatine kinase, alkaline phosphatase) to protect bone tissue (54, 55); (c) At the neurohumoral regulation level, acupuncture stimulation of the abundant superficial nerve endings triggers neurohumoral responses. Beyond promoting endorphin release, this stimulation relieves muscle spasms, improves the biomechanical function of affected joints, and ultimately enhances patients’ motor capacity and quality of life (56).

Furthermore, distinct acupuncture modalities exhibit targeted unique mechanisms (57): (a) Warm needle acupuncture: Combining thermal stimulation with needling, it improves local circulation, inhibits inflammatory mediators, and reduces IL-17 levels. This blocks the “IL-17/IL-23 mediated upregulation of calcium-sensing receptor (CaSR) in osteoblasts” pathway, thereby delaying pathological ossification in AS (58); (b) Floating needling: By sweeping subcutaneous connective tissue, it generates bioelectrical signals along fascial planes, rapidly altering cell signaling and membrane permeability in target regions to achieve rapid pain relief (59, 60); (c) Fire needling: Exerting both mechanical and thermal stimulation at acupoints, it warms and unblocks meridians, activates blood circulation to resolve stasis, and alleviates pain and stiffness (43); (d) Acupotomy: Performs microincisions at tender points to regulate pain-related neuropeptides (e.g., increasing substance P levels), exerting therapeutic effects through neurochemical changes (49, 50, 61, 62).

Given the complex systemic nature of AS, multimodal treatment strategies integrating internal and external therapies may offer more comprehensive symptom control. An integrated strategy combining internal therapies (e.g., systemic medications) and external therapies (e.g., acupuncture, physical therapy) may provide the most effective management. In TCM, internal medicine (herbal therapy) and external treatment (acupuncture) have complementary strengths (63). Combining them may produce synergistic effects: acupuncture can enhance the distribution and efficacy of medications (helping them reach the entire body) (28). In contrast, medications can prolong or amplify the benefits of acupuncture in unblocking meridians (26). By targeting both internal pathology and external symptoms, this combined approach could yield superior outcomes in AS (27).

Despite these encouraging findings, several limitations warrant consideration. The included trials differed in acupuncture techniques, practitioner expertise, and acupoint selection, which may have contributed to heterogeneity in treatment effects. Moreover, the overall methodological quality was moderate, with frequent issues such as lack of blinding and small sample sizes, limiting the robustness of the conclusions. Most outcomes relied on patient-reported measures, while objective indicators such as imaging or biomarkers were seldom employed. In addition, the underlying mechanisms of AS and of acupuncture’s therapeutic effects remain insufficiently understood, and certain outcome measures required standardization or data approximation during synthesis, potentially introducing minor errors.

Future research should prioritize the establishment of standardized acupuncture protocols, which would help reduce heterogeneity and enable more reliable comparisons across studies. Equally important is the conduct of high-quality, multicenter randomized controlled trials with rigorous methodological safeguards, such as adequate blinding and appropriate sample sizes, to strengthen the evidence base. The use of more objective outcome measures, including imaging techniques and functional assessments, alongside patient-reported scales, could improve the accuracy of efficacy evaluation and minimize bias. Furthermore, long-term follow-up studies are needed to clarify not only the sustained effects of acupuncture and moxibustion but also their potential influence on disease progression and overall prognosis.

Nevertheless, this study still holds great significance. Through meta-analysis, this study found that in addition to conventional needling, specialized acupuncture modalities such as fire needling, catgut embedding, and acupotomy also serve as effective supplementary treatments for AS. Furthermore, no acupuncture-related serious adverse events were reported in the trials included in this study, indicating that acupuncture has good safety as an adjuvant therapy. Currently, recommendations on acupuncture for AS treatment remain relatively scarce in international guidelines (64). This study provides quantitative evidence for the adjuvant role of acupuncture (including specialized modalities) in improving AS symptoms, which is expected to offer a reference basis for the development and update of relevant guidelines in the future. Although this study still lacks sufficient evidence to directly compare the efficacy of the aforementioned different acupuncture techniques, which makes it impossible to objectively rank their effectiveness, it also points out a direction for subsequent research.

In the future, we hope that more researchers will build on these findings by expanding research in this field and by developing more standardized acupuncture protocols, thereby providing stronger and more comparable evidence for integrating acupuncture into the clinical management of AS and related immune diseases.

## Conclusion

As revealed in this systematic review, acupuncture (including electroacupuncture, warm acupuncture, and fire acupuncture) is effective and safe for AS, for it not only markedly reduces scores on scales like BASFI, BASDAI, ASDAS, and VAS (thereby alleviating back pain and improving disease activity and functional status) but also lowers CRP and ESR levels to curb inflammation. Rarely reported adverse events, coupled with its advantages of being eco-friendly, cost-effective, and efficacious, further highlight its level. However, significant heterogeneity across studies, combined with the paucity of large-scale, high-quality controlled trials, undermines the current evidence base. Thus, future research should prioritize high-quality multi-center trials and enhanced comparisons between therapies to objectively evaluate various acupuncture approaches and refine the evidence system for acupuncture in AS treatment.

## Data Availability

The original contributions presented in the study are included in the article/Supplementary material, further inquiries can be directed to the corresponding author.
