# Supplementary material for: Efficacy of acupuncture in the management of ankylosing spondylitis: a systematic review and meta-analysis with insights
Source: Front Neurol. 2026 Jan 13;16:1716550. doi: 10.3389/fneur.2025.1716550 (PMC12836302; doi:10.3389/fneur.2025.1716550)
Supplement: Supplementary file 1 [file Data_Sheet_1.docx]

Supplementary Material

Efficacy of Acupuncture in the Management of Ankylosing Spondylitis: A Systematic Review and Meta-Analysis with Insights

**Junning Zhang^1,2^†, Shuchang Sun^3^†, Ruitong Bai^4^, Hechun Yin^1,4^, Jiawen Chen^1,2^ and Weiping Kong^2^***

^1^ Graduate School, Beijing University of Chinese Medicine, Beijing, China

^2^ Department of Traditional Chinese Medicine Rheumatology, China-Japan Friendship Hospital, Beijing, China

^3^ School of Acupuncture-Moxibustion and Tuina, Beijing University of Chinese Medicine, Beijing, China

^4^ Dongfang Hospital, Beijing University of Chinese Medicine, Beijing, China

† These authors contributed equally to this work.

*** Correspondence: Weiping Kong***

drkongweiping@163.com


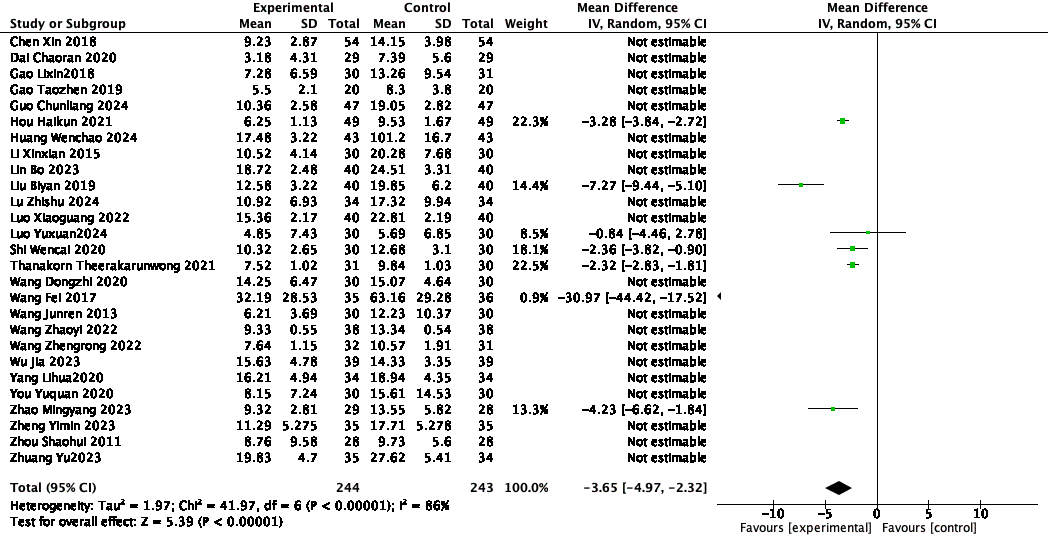


Subgroup Analysis: Forest Plot of Acupuncture Intervention in Subgroup Analysis for Ankylosing Spondylitis


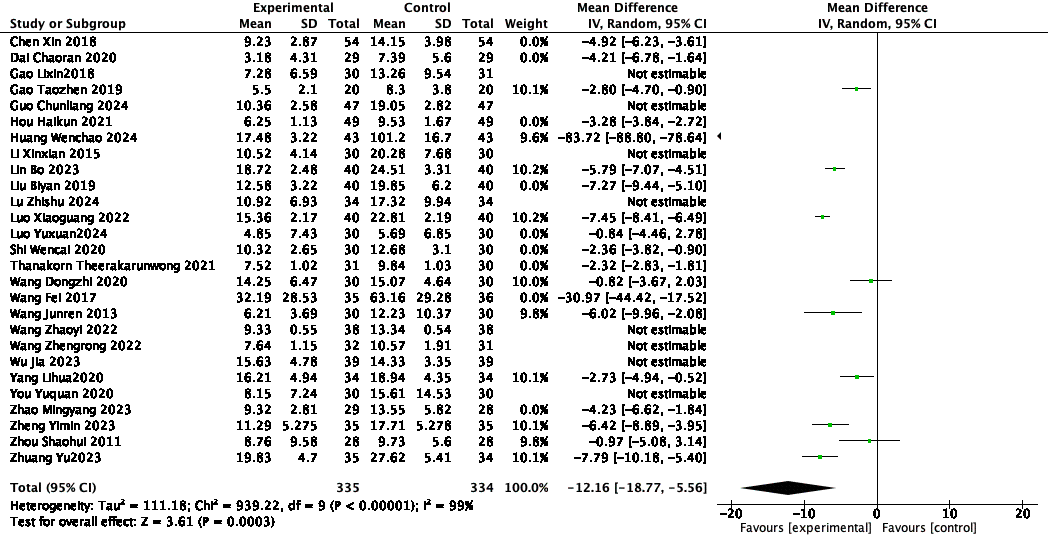


Subgroup Analysis: Forest Plot of Warm Acupuncture Intervention in Subgroup Analysis for Ankylosing Spondylitis


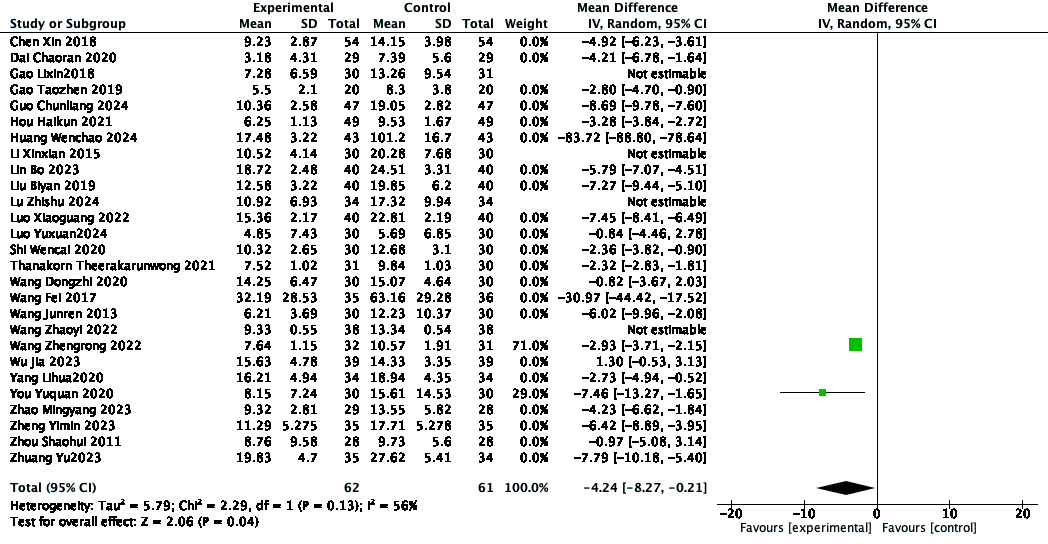


Subgroup Analysis: Forest Plot of Needle-Knife Therapy Intervention in Subgroup Analysis for Ankylosing Spondylitis


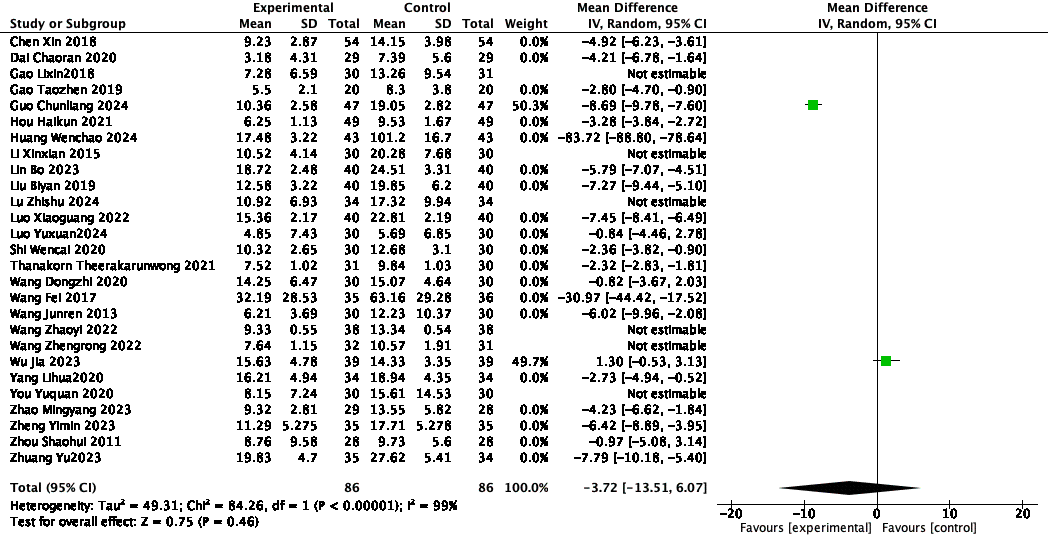


Subgroup Analysis: Forest Plot of Exercise-Acupuncture Intervention in Subgroup Analysis for Ankylosing Spondylitis


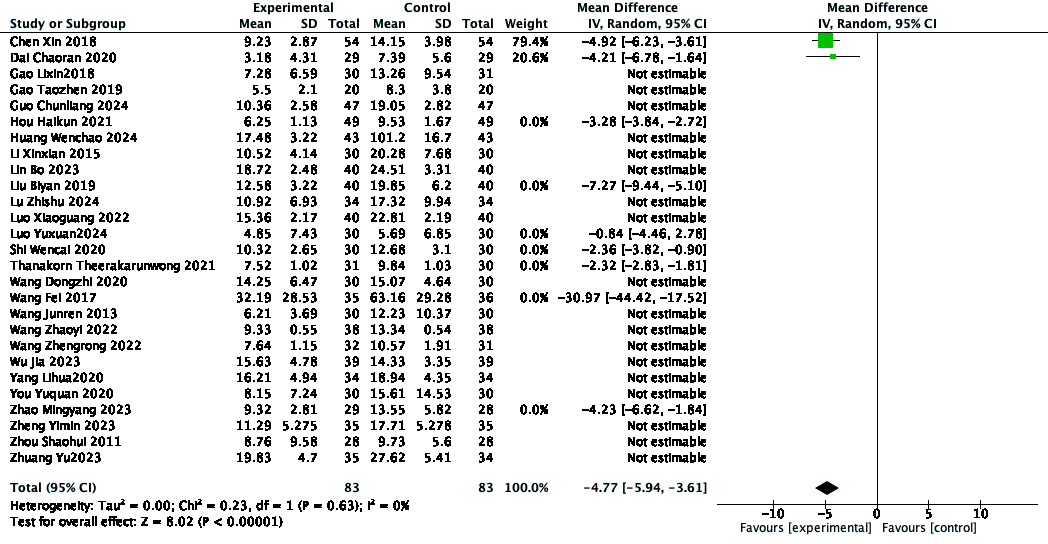


Subgroup Analysis: Forest Plot of Electroacupuncture Intervention in Subgroup Analysis for Ankylosing Spondylitis


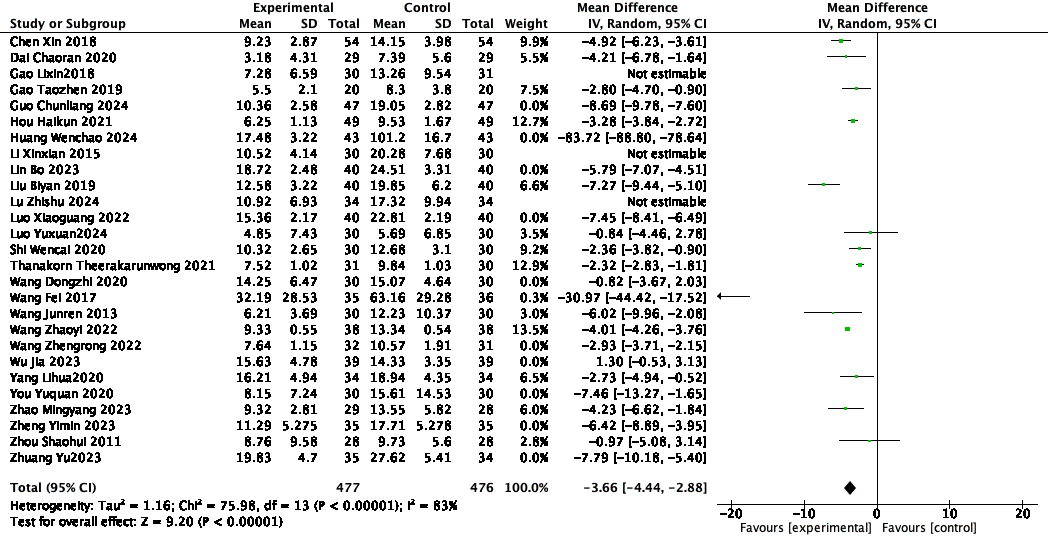


Subgroup Analysis: Forest Plot for Ankylosing Spondylitis - 30-minute Intervention


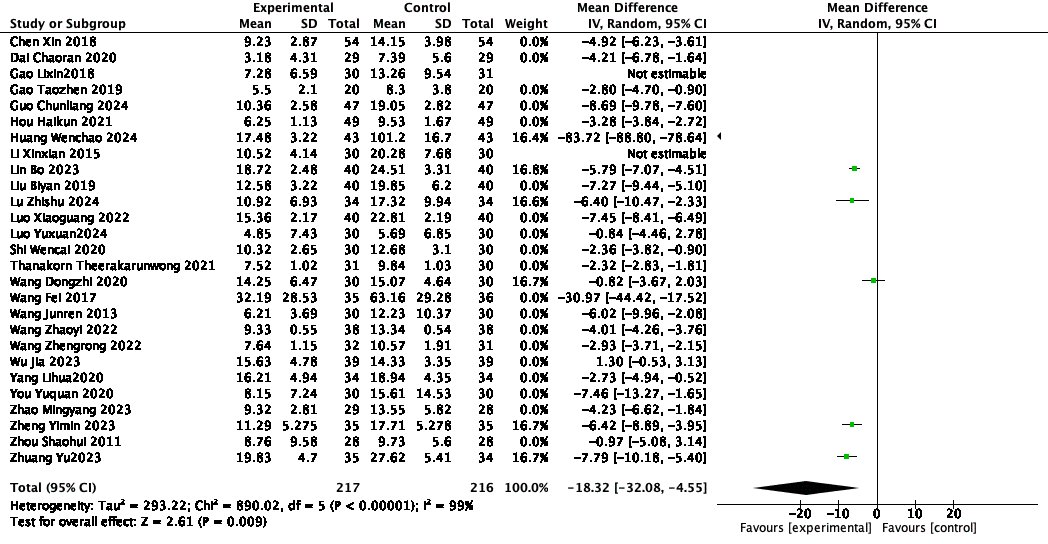


Subgroup Analysis: Forest Plot for Ankylosing Spondylitis - Intervention Time More Than 30 Minutes


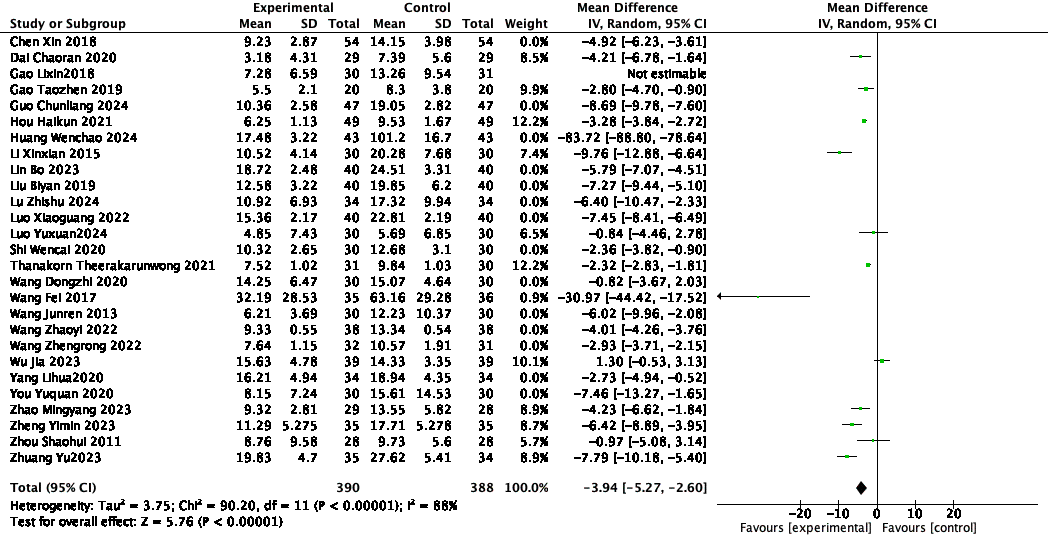


Subgroup Analysis: Forest Plot for Ankylosing Spondylitis - Intervention Duration Less Than 5 Weeks


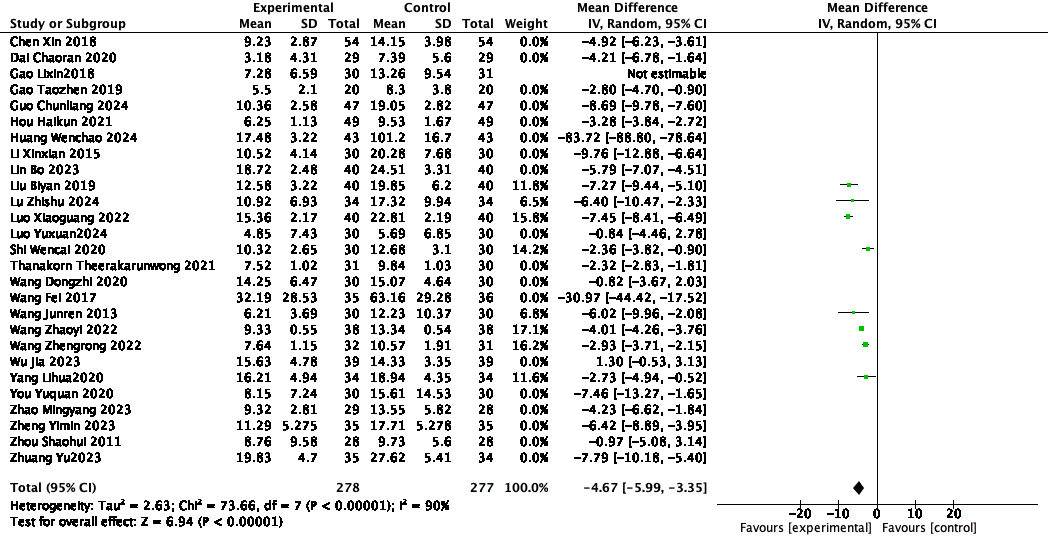


Subgroup Analysis: Forest Plot for Ankylosing Spondylitis - Intervention Duration 5 Weeks to 10 Weeks


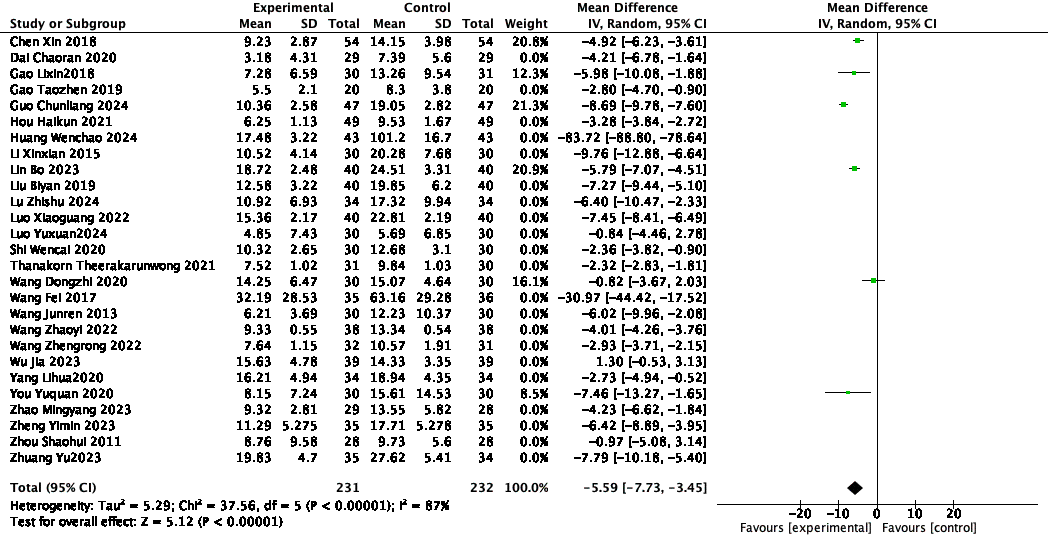


Subgroup Analysis: Forest Plot for Ankylosing Spondylitis - Intervention Duration More Than 10 Weeks
